# Feasibility and acceptability of ENhanced Reviews of PsychologIcal Changes (ENRICH) after stroke: protocol

**DOI:** 10.1136/bmjopen-2026-116566

**Published:** 2026-05-24

**Authors:** Andrea Kusec, Georgina Hobden, Alexandra Wright-Hughes, Nele Demeyere

**Affiliations:** 1 Nuffield Department of Clinical Neurosciences, University of Oxford, Oxford, UK; 2 Department of Experimental Psychology, University of Oxford, Oxford, UK; 3 Clinical Trials Research Unit, University of Leeds, Leeds, UK

**Keywords:** Stroke, QUALITATIVE RESEARCH, Feasibility Studies, Clinical Protocols, Cognition, Depression & mood disorders

## Abstract

**Introduction:**

Managing mood, cognition and fatigue are top unmet needs reported by stroke survivors, which impact quality of life. There is currently no standardised UK care pathway to support post-stroke psychological outcomes. The ENhanced Reviews of PsychologIcal Changes (ENRICH) programme, an intervention co-designed with stroke survivors, carers and healthcare professionals, aims to fill this gap. Here, we describe the protocol for evaluating the feasibility and acceptability of ENRICH.

**Methods and analysis:**

ENRICH reviews comprise cognition, mood and fatigue assessment, personalised psychoeducation and tools to communicate results and discuss self-management strategies, delivered at 1, 3 and 6 months post-stroke. N=140 participants (N=80 patients who had a stroke, N=45 carers, N=15 healthcare professionals) will be recruited to a single-arm multicentre feasibility study.

Patients who had a stroke and carers will complete demographics at baseline (T1) questionnaires of quality of life, mood and healthcare resource use at 6 months post-stroke (T2) and an optional interview on experiences of ENRICH. Process evaluation will include fidelity assessment via audio recordings. Descriptive statistics will be calculated for study outcomes.

Key qualitative acceptability outcomes are sought on intervention delivery by clinicians, patients and carers.

Key intervention delivery feasibility outcomes relate to training clinicians (including competency and fidelity delivering ENRICH), and review completion rates. Study feasibility outcomes will include site and participant recruitment and retention rates and completion of candidate primary outcome measures on quality of life.

**Ethics and dissemination:**

The ENRICH study was approved by a UK Research Ethics Committee (reference: 24/LO/0341). Consent procedures include a waiver of consent to the intervention itself due ENRICH’s service-level design and written informed consent/consultee advice for participants providing research data. Results will be disseminated through peer-reviewed publications, conferences and lay summaries for study participants and healthcare professionals. Results will inform whether ENRICH is acceptable to delivering clinicians and receiving patients who had a stroke and carers, and provide key insights to inform a future randomised trial to determine effectiveness.

**Trial registration number:**

ISRCTN16018388.

**STRENGTHS AND LIMITATIONS OF THIS STUDY**

- Mixed-methods design: This study uses both quantitative and qualitative data to comprehensively assess key uncertainties in the feasibility study to capture both depth and breadth.
- Fidelity monitoring: We will capture adherence to the ENhanced Reviews of PsychologIcal Changes (ENRICH) protocol in depth via self-reported fidelity checklists and audio recordings of intervention sessions.
- Patient and public involvement: We have thoroughly reviewed study materials and selected candidate primary outcome measures with input from those with lived experience of stroke and their family members.
- No control arm: Due to prioritising information about how the novel, co-produced intervention is delivered in sites, we have used a single-arm multicentre feasibility study design.
- No randomisation: Participants are not randomised to receive the ENRICH intervention.

## Introduction

 Stroke is a chronic neurological condition that is the number one cause of disability globally.^1^ The majority of stroke survivors experience psychological changes such as cognitive impairment, depression, anxiety and fatigue. Post-stroke cognitive impairment can affect domains of memory, attention, language, executive functioning and number processing abilities, with 6-month prevalence of any type of cognitive impairment as high as 68%.^2^ Post-stroke depression and anxiety are also common, affecting roughly 25–30% of stroke survivors.^3 4^ Finally, post-stroke fatigue rates reach up to 50% in terms of prevalence.^5^

Screening for post-stroke psychological changes, such as mood and cognition, is now recommended by national and international clinical guidelines for stroke.^6^ However, managing mood and cognitive changes are the top two unmet needs following stroke, alongside other psychological outcomes.^7^ Further, 81.4% of stroke survivors and carers report wanting more information on managing and understanding psychological changes after stroke^8^ with clearly evidenced psychoeducation needs on post-stroke cognition.^9^ Despite improvements in the first 6 months post-stroke,^10^ psychological changes can persist chronically, with prevalence rates long-term post stroke ranging from 25% to 50%.^11^

Providing ongoing support for psychological changes is especially important because these changes have a substantial impact on post-stroke quality of life.^2 11^ Importantly, research on the topics of post-stroke mood and cognitive changes was ranked as the top two priorities within the most recent James Lind Alliance priority setting exercise.^7^ In the UK, the National Health Service (NHS) recommends that all patients who had a stroke are offered a single 6-month review of their recovery and abilities following discharge from acute/subacute stroke services. However, the current stroke care pathway does not routinely provide care for recognising, understanding and managing post-stroke psychological outcomes, with many reviews primarily focused on secondary stroke prevention or on physical stroke recovery (eg, blood pressure monitoring, medication review^12^). This is in part due to a lack of sufficient training on how to screen for and communicate post-stroke psychological outcomes and inconsistency in post-stroke reviews provided across the UK.

The ENhanced Reviews of PsychologIcal Changes (ENRICH) intervention was developed iteratively and rigorously following the Medical Research Council (MRC) framework for complex intervention development,^13^ detailed in Hobden and Demeyere.^14^ The final ENRICH intervention consists of an iterative (1, 3 and 6 months) monitoring and management programme for post-stroke psychological changes using ENRICH reviews. Each ENRICH review provides assessment, feedback and personalised psychoeducation on domain-specific cognition, mood and fatigue, with self-management strategies and onward referral to specialist services as indicated. The ENRICH training and reviews also emphasise non-specific elements of psychological care, such as personalisation, explaining jargon, fostering hope and optimism and using compassion and sensitivity throughout the reviews. The ENRICH intervention provides a holistic framework for enhancing the psychological care pathway for stroke survivors.

In this study, we aim to determine the feasibility, acceptability and fidelity of ENRICH reviews delivered in clinical stroke services by a range of healthcare professionals to inform a potential future pragmatic randomised cluster trial.

## Methods and analysis

### Design

The ENRICH feasibility study is a single arm multicentre feasibility study across two sites, delivered at the site/cluster level, capturing detailed quantitative and qualitative data on feasibility, acceptability and fidelity focused primarily on the ENRICH intervention itself, aimed at informing the design of a future pragmatic cluster randomised controlled trial. We considered a site to be at least one NHS stroke service comprising the full stroke care pathway from acute to 6 months post-stroke. Where this comprised two separate services, this was considered one site for the purposes of delivering the ENRICH intervention.

#### Our specific aims were to evaluate

1. Acceptability of ENRICH to NHS healthcare professionals, patients who had a stroke and family members, and of quality of life and health economic outcome measures.
2. Feasibility of site recruitment, participant recruitment and retention rates, most effective methods of consent and delivery of ENRICH reviews.
3. Fidelity of the ENRICH intervention when delivered by NHS healthcare professionals.

All recruited sites will only be required to implement the ENRICH intervention (ie, no control site). Given data in acute and subacute stroke populations on recruiting and conducting complex interventions at the cluster level using controlled designs,^15^,^17^ we have adopted a design to capture the most relevant data and prioritise evaluation of ENRICH intervention training and delivery, to inform progression to a pragmatic effectiveness trial. We include a waiver of consent for the ENRICH intervention delivery only in accordance with the Ottawa statement on Ethical Design and Conduct of Cluster Randomised Trials,^18^ given that the intervention is arguably in line with minimum stroke care standards and is unlikely to cause harm. The waiver of consent covers the delivery of the full ENRICH intervention delivered at 1, 3 and 6 months post-stroke, including conducting the assessments, communicating screening results and self-management of psychological changes (see ENRICH intervention below for further specifics). A single-arm multicentre feasibility study design with a waiver of consent for intervention delivery was selected because (1) ENRICH was designed to be implemented for all patients who had a stroke in a service as a standard of psychological care; (2) we aim to evaluate the ENRICH intervention as a service change for all, rather than an intervention for only a specific subset of patients who had a stroke, and (3) there was deemed a potential for contamination within a service where ENRICH-trained healthcare professionals typically jointly discuss patients who had a stroke care, thereby also limiting the potential for evaluation within an individually randomised controlled trial.

The waiver of consent does not cover transfer of any research data about the ENRICH assessments or identifiable patient demographics to the research team outside of the patient’s NHS service. Written informed consent or favourable consultee advice is required to be in place for this research data collection to occur. Only fully anonymised site-level information will be collected about total number of patients in the recruited site who had received an ENRICH review during the study time frame. Patients lacking capacity will be protected through having consultee advice procedures in place, conducting with a family member who knows the patient well, to advise on what they believe the patient would have wanted with regards to research data collection.

We set out to evaluate context-specific uncertainties prior to an effectiveness trial to maximise usefulness of information gained, in line with the MRC complex intervention framework for feasibility studies.^13^ The ENRICH intervention was envisioned as a service change, with the ultimate implementation aim to deliver enhanced reviews to all patients who had a stroke as a new Level 2 standard^19^ of post-stroke psychological care in line with clinical recommendations.^20^ Here, we closely aligned the intervention development with consensus views on what psychological care should look like^21^ and aimed to evaluate this complex intervention through a future implementation lens in line with integrating implementation goals closely within the complex intervention evaluation.

The ENRICH key uncertainties, as identified with stakeholder involvement, related mainly to study design, intervention delivery and research data collection elements.

#### Uncertainties in Study Design

Given the focus on intervention delivery in the first 6 months post-stroke where active care is still present, it was unknown how many sites would be eligible (see Participants section for inclusion/exclusion criteria) and interested in taking part in ENRICH. Further, it was unknown if during this point of active care delivery whether sufficient patients who had a stroke and family members numbers could be recruited to research data collection, and whether the recruited stroke sample was comparable to non-consenting clinical populations (eg, in terms of stroke severity, degree of cognitive impairment).

Uncertainty exists on whether a future randomised cluster design, with a waiver of consent to the intervention itself, would be feasible in terms of number of reviews delivered to non-consenting patients who had a stroke, and to what degree each consenting method (standard consent, consultee advice) would be used throughout the trial. Finally, given the wide variation in how psychological stroke care is provided across services, it is unknown what the minimum care standard for a future potential control arm would look like.

### Uncertainties in Intervention Delivery

Given that ENRICH is a novel, co-produced intervention, the acceptability of the reviews by patients who had a stroke, family members and healthcare professionals is unknown. Second, as ENRICH was designed to be delivered over an extended time frame post-stroke by members of the stroke care team, the feasibility of training a variety of clinicians to deliver the ENRICH reviews with high adherence to the key intervention requirements was identified as another key uncertainty.

### Uncertainties in Research Data Collection

It was unknown what the estimated completion rates and attrition would be for completion of the outcome measures, as well as uncertainty around which of two candidate quality of life outcome measures to use and whether health economic data could be captured adequately (see Outcomes for details on study measures). Further, it was unknown whether participants would find collecting fidelity data (see Fidelity section below) acceptable.

Taken together, we aimed to investigate acceptability and feasibility of the intervention delivery, as well as design feasibility of a future cluster design including waiver of consent for delivery of the ENRICH intervention. A summary of our key uncertainties and associated data we aim to collect is in table 1. Our qualitative aims with regards to delivering the intervention were to explore the acceptability of the ENRICH training by healthcare professionals and the ENRICH reviews by patients who had a stroke, carers and healthcare professionals through surveys and interviews. In addition, we will examine the fidelity to non-specific factors through audio recordings of reviews and explore barriers and facilitators of the implementation of the ENRICH reviews in clinical stroke services. With regards to study design, we aim to explore participants’ views on study procedures, materials and outcome measures, alongside understanding standard clinical care.

**Table 1:** Summary of key uncertainties and associated data to be collected

Evaluation domain	Study component	Data source	Key uncertainty	Associated quantitative data	Associated qualitative data
**Feasibility**	**Study design**	Sites	Can we recruit sites?	Number of eligible sites expressing interest	
			How many patients receive ENRICH with waiver of consent?	Total number of reviews delivered	
			What is the standard of care?	Number of non-ENRICH clinical contacts	Descriptions of standard care across each recruited site
		Healthcare professionals	What is the most effective consent method?	Proportion consented by clinician role	
			Can we recruit and retain sufficient numbers?	Recruitment and attrition rates	
		Patients who had a stroke	Can we recruit and retain sufficient numbers?	Recruitment and attrition rates	
			Can we recruit a diverse sample?	Patient demographics vs national audit data	
			How many ENRICH reviews do participants receive?	ENRICH review completion rates	Reasons for non-completion
		Family members	Can we recruit and retain sufficient numbers?	Recruitment and attrition rates	
Acceptability	Intervention delivery	Healthcare professionals	Can we train a variety of healthcare professionals to deliver ENRICH?	Numbers trained per site	Training survey feedback and interviews
			What are their views on ENRICH?	Helpfulness and usefulness ratings of ENRICH	Interviews on barriers and facilitators to ENRICH
			How many of those trained deliver the intervention?	Proportion of those trained delivering ENRICH	
		Patients who had a stroke	What are their views on ENRICH?	Helpfulness and usefulness ratings of ENRICH	Interviews on acceptability of ENRICH
		Family members	What are their views on ENRICH?	Helpfulness and usefulness ratings of ENRICH	Interviews on acceptability of ENRICH
	Research data collection	Patients who had a stroke and Site principal investigators	Which candidate primary outcome measure is best to use?	QOLIBRI and WHO-QOL-BREF completion rates	Interviews on acceptability of QOLIBRI and WHO-QOL-BREF
			Can we collect health economic information?	HEQ completion rates	
			Can we collect process outcome measures?	Process measure completion rates	
			Can we collect audio recorded fidelity data?	Proportion consenting to audio recordings	
Fidelity	Intervention delivery	Healthcare professionals	Are ENRICH intervention components delivered with high fidelity?	Completion checklists of intervention components; ratings of intervention audio recordings	

ENRICH, ENhanced Reviews of PsychologIcal Changes; HEQ, Health Economics Questionnaire; QOLIBRI, Quality of Life Instrument after Brain Injury; WHO-QOL-BREF, WHO-Quality of Life-Brief.

### Patient and public involvement

A patient carer and public involvement (PCPI) committee of five stroke survivors and two family members provides oversight into study procedures, study materials review, patient recruitment and support site set-up and training of healthcare professionals. The PCPI committee meets quarterly for the duration of the study. Prior to finalising the study design, the PCPI committee selected the candidate primary outcome measures, finalised participant-facing materials and design of fidelity scoring materials.

### Setting

The study will take place in England, UK, including stroke services that span the full care pathway from acute hospitalisation to 6-month review (see table 2 for details on eligible sites).

**Table 2:** Inclusion and exclusion criteria of study participants

Sites	Patients who had a stroke	Carers	Healthcare professionals
Inclusion criteria			
Existing 6-month review offered to all patients who had a stroke within the service	Aged 18 years or older	Aged 18 years or older	Currently working within eligible stroke pathway NHS sites
Existing early supported discharge stroke service within NHS England	Within 4 weeks of confirmed clinical diagnosis of stroke (first ever or recurrent)	Cohabiting with stroke survivor	Currently deliver stroke reviews within eligible stroke pathway NHS sites
Capacity for research activity within the clinical team (ie, capacity for recruitment and to conduct enhanced reviews)	Sufficient English fluency	Able and willing to provide informed consent	Are able and willing to provide informed consent
	Ability to concentrate for 10 min, as judged by the clinical team		
	Ability to provide informed consent or favourable consultee advice for adults lacking capacity to consent		
Exclusion criteria			
		They are a paid or professional carer	

NHS, National Health Service.

### Participants

Patients who had a stroke, carers and healthcare professionals will be recruited. For patients who had a stroke, intentionally broad inclusion criteria (table 2) are used to ensure representativeness of real-world stroke clinical settings. Each stroke pathway will be responsible for the identification and recruitment of patients who had a stroke and carer participants and the delivery of ENRICH reviews.

Recruitment will occur following site initiation and training. Patients who had a stroke will be screened for eligibility within recruited sites and approached for participation by multidisciplinary team members. Given the waiver of consent for intervention delivery, recruited stroke participants will be invited to provide informed consent to provide outcome data collection and an optional semi-structured interview (see Outcomes for further details). Patients who had a stroke will be asked to optionally nominate a carer who the multidisciplinary team could approach for participation; not having a carer or not wishing a carer to take part will not preclude participation. Nominated carers will then be approached by the multidisciplinary team to take part and provide informed consent.

Clinical leads at each recruited site will identify healthcare professionals who meet study eligibility criteria, and will be approached following site initiation about participation. All study data collected will be held confidential and only accessible by those in the research team, with data made fully anonymised past study end.

### Healthcare professional training

The ENRICH intervention is intended to be a Level 2 psychological care intervention, in line with guidelines on providing matched care to stroke survivors,^19^ that can be provided by any member of a multidisciplinary team. Healthcare professionals at recruited stroke services are provided with 4 hours of training (in person or remotely via videoconferencing offered) covering an overview of the ENRICH study purpose and intervention structure, practical use of the ENRICH materials (eg, assessments, ENRICH patient workbook) and role play activities to familiarise themselves with implementation of ENRICH reviews.

### ENRICH Intervention

The ENRICH intervention provides stroke survivors with structured psychological reviews and 1, 3 and 6 months post-stroke, aiming to extend standard UK stroke reviews delivered at 6 months post-stroke only. In accordance with our co-production work^14^ ENRICH reviews comprise screening of psychological outcomes using standardised assessments of objective (Oxford Cognitive Screen; OCS^22^) and subjective (Checklist for Cognitive, Emotional and Behavioural Consequences following Stroke; CLCE^23^), mood (Patient Health Questionnaire-9; PHQ-9^24^), anxiety (Generalised Anxiety Disorder-7; GAD-7^25^) and fatigue (Fatigue Severity Scale; FSS).^26^ ENRICH reviews include communicating screening results from these measures and their implications to patients who had a stroke, and psychoeducation and self-management about their specific psychological changes. Where the stroke survivor views it to be beneficial, family members of stroke survivors are also invited to attend ENRICH reviews and complete a measure of subjective cognition of the stroke survivor to contribute to the review process. These elements are scaffolded using a co-designed ENRICH workbook, meant to serve as a communication tool and record of screening outcomes and information relevant to the patients who had a stroke. In addition to these specific factors, the co-production of ENRICH highlighted that reviews should also incorporate non-specific factors in terms of how each ENRICH component is implemented. ENRICH reviews should therefore include personalisation of reviews to the patients who had a stroke’s needs and circumstances, use accessible language and explaining any jargon terms, foster hope and optimism in the patients who had stroke and use compassion and sensitivity throughout the reviews. ENRICH reviews are designed to be delivered by clinicians providing Level 2 care in stroke services, including (but not limited to) assistant psychologists, occupational therapists, therapy assistants and speech language therapists.

In our single-arm multicentre feasibility study design, all patients who had a stroke in the site can receive ENRICH. We aim for all consenting and those with favourable consultee advice to receive ENRICH reviews. Participants will not be required to discontinue any ongoing care while receiving ENRICH.

### Participant Timeline

Stroke participant demographic data will be collected prior to the first ENRICH review at baseline (T1). Outcome data will be collected either via post, live via telephone/videoconferencing or using online surveys following completion of the final enhanced review at 6 months after stroke (T2; see table 3 for overview). Candidate outcomes were not collected at baseline as self-report assessments of quality of life were viewed not to be representative at time post-stroke of high emotional distress.

**Table 3:** Overview of patients who had a stroke study outcome measures and additional data

	Baseline (T1)	Post-ENRICH assessment (T2)
Demographic information	✔	
Secondary outcome measures—candidate primary outcome measures for a future trial		
Quality of Life Instrument after Brain Injury		✔
WHO-Quality of Life Brief		✔
Process measures		
Patient Health Questionnaire-9		✔
Generalised Anxiety Disorder-7		✔
Modified Rankin Scale		✔
EQ-5D-5L		✔
Health Economics Questionnaire		✔
Additional data collection		
Custom post-study survey		✔
Semi-structured interview (~30% of participants)		✔

ENRICH, ENhanced Reviews of PsychologIcal Changes; EQ-5D-5L, EuroQol-5D-5Levels.

### Quantitative Outcome Measures

As family members/carers and healthcare professionals will only provide qualitative research data through a semi-structured interview and custom survey data, the below outcome measures will be completed by patients who had a stroke consenting to research data collection only.

### Primary Outcome

Given the study aims, the primary outcome measures for this study are feasibility measured by descriptive statistics on recruitment, attendance and completion of study measures over the course of the study.

### Key Secondary Outcome Measures—Candidate Primary Outcome Measures for a Future Trial

In order to ultimately evaluate whether ENRICH is an effective intervention, a future effectiveness trial should evaluate a meaningful outcome. In collaboration with our PCPI group, the candidate primary outcome for future evaluation was determined to be quality of life. In the present study, these are key secondary outcome measures relative to the primary outcome measures assessing feasibility above. Following PCPI discussions, we selected two potential candidate primary outcome measures as below, for which we aimed to determine acceptability and completion rates in the present study.

The Quality of Life Instrument after Brain Injury (QOLIBRI) is a 37-item self-report measure of health-related quality of life following brain injury. The measure is designed to measure aspects of health-related quality of life associated specifically with brain injury such as thinking abilities, emotions, functioning and social activities and the degree to which individuals are bothered by them. The QOLIBRI was specifically developed and validated for brain injury populations.^27^

The WHO-Quality of Life-Brief (WHO-QOL-BREF) is a 39-item patient-reported outcome measure designed to capture quality of life across physical, psychological and environmental domains and capture how quality of life has been affected by disability. The WHO-QOL-BREF has been validated for patient use across multiple patient populations including stroke.^28^

### Process Measures

Process measures were selected to capture theoretically relevant constructs that may moderate responses to the ENRICH intervention or shift as a result of the intervention, as well as provide descriptive information about improvement more generally. This includes depressive symptoms (PHQ-9), anxiety symptoms (GAD-7) and functional ability (modified Rankin Scale^29^).

### Additional Quantitative Data Collection

We will additionally collect scores on the OCS, CLCE, PHQ-9, GAD-7 and FSS completed with patients who had a stroke during ENRICH reviews. These will be used to describe the recruited cohort.

### Health Economic Evaluation

Alongside candidate primary and secondary outcome measures, stroke participants will complete measures of healthcare resource use and quality of life including the EuroQol-5D-5Levels and the Health Economics Questionnaire (HEQ) is a 5-item customised measure of health resource use post-stroke, developed with a health economist and the PCPI committee. Participants are asked to report on amount and type of unpaid care received, medication use, re-admission to hospitals, health service use (eg, General Practitioner use) and employment status.

### Quantitative Data for Uncertainties in Study Design

With regards to study design, quantitative data on number of sites meeting initial site eligibility and numbers of expressions of interest will be collected. Per site, participant eligibility rates (defined as percentage of patients per site meeting study eligibility criteria), recruitment rates (total number of patients recruited per month across both informed and consultee advised consent), intervention completion (total number of ENRICH reviews received) and outcome completion rates for patients who had a stroke, carers (T2 assessment completion rates) and healthcare professionals (interview completion) will be collected.

### Quantitative Data for Uncertainties in Intervention Delivery

Our quantitative aims with regards to the delivery of the intervention are to investigate the numbers of trained clinicians delivering ENRICH reviews, proportions of recruited patients receiving ENRICH reviews and ratings of perceived helpfulness and usefulness (assessed using a custom Likert scale from 1 to 5) of ENRICH from healthcare professionals, patients who had a stroke and carers.

### Quantitative Data for Uncertainties in Research Data Collection

We aim to assess completion rates of candidate outcome measures (ie, total proportion of participants completing each outcome measure) at T2 to inform acceptability. We will additionally assess total proportion of patients consenting to audio recordings for fidelity. Through the HEQ, we will additionally assess number of healthcare services used to describe usual care and inform the future control arm.

### Qualitative Evaluation

A key aim of this study is to determine the acceptability and feasibility of the intervention delivery and evaluate whether and how this delivery could work as a service-level care pathway intervention for a future cluster randomised study. Therefore, the qualitative evaluation by both clinicians delivering the intervention as well as participants and carers receiving the intervention is key to assessing acceptability of intervention delivery. All interviews will be audio recorded with consent and transcribed verbatim.

### Qualitative Data for Uncertainties in Study Design

Reasons for non-completion of ENRICH reviews will be collected to inform the source of non-delivery of ENRICH reviews (eg, no time from clinical staff, participant refusal).

### Qualitative Data for Uncertainties in Intervention Delivery

Consenting healthcare professional participants will be invited to complete a custom survey and semi-structured interview about their feedback on contributing to ENRICH. The interview will focus both on barriers and facilitators to implementing ENRICH with patients who had a stroke and identify elements to maximise implementation of ENRICH reviews at the service level. We will probe for feedback on refining the ENRICH training for future healthcare professionals, experiences conducting ENRICH assessments and using the ENRICH workbook and how ENRICH is adopted into practice by those involved. We will aim to interview all consenting healthcare professionals and site principal investigators to maximise information gained.

Patients who had a stroke and carer participants consenting to research data collection will be invited to complete a custom post-study survey about their experience of ENRICH reviews at T2. In addition, a subset of purposively sampled stroke participants will be invited to complete a semi-structured interview on their experience of ENRICH reviews and study procedures. The topic guide will probe general experiences receiving ENRICH reviews, aspects that were helpful and not helpful, feedback on assessments used during reviews and using the ENRICH workbook. Carers will be invited to complete a semi-structured interview of their experience of ENRICH reviews and/or their perspective of the stroke survivor’s experience of ENRICH reviews, where we will probe their views on its helpfulness to the stroke survivor, the role of the family member in ENRICH reviews and its benefit beyond the review sessions themselves. Across all patients who had a stroke interviews, maximum variation sampling will be used to select a range of stroke severity, type of cognitive impairments (eg, language, memory, attention), participant sex, ethnicity and self-reported mood to enhance richness of the data and inform conclusions about acceptability.

### Qualitative Data for Uncertainties in Research Data Collection

In interviews, we will explore the acceptability of the candidate primary outcome measures (QOLIBRI and WHO-QOL-BREF) and the health economic measure (HEQ) given limited use of these measures in stroke. We will also explore acceptability of reviews being recorded for fidelity purposes.

From recruited sites, we will collect surveys on descriptions of usual care to inform what a future control arm would look like.

### Fidelity Data

As part of a process evaluation, fidelity to the intervention will be evaluated in two ways:

1. Healthcare professional-reported fidelity checklists of key components delivered of each ENRICH review.
2. Audio recorded ENRICH reviews conducted with consenting stroke participants.

The above will inform whether the ENRICH intervention was delivered as intended and whether sufficient adherence can be achieved across recruited healthcare professionals.

The research team will also hold monthly meetings with local site champions (eg, clinical lead, principal investigator or senior therapists for service) and available healthcare professionals delivering the enhanced reviews to discuss any difficulties with implementation. Daily problem solving, if any, will be conducted in discussion with the principal investigator of recruited NHS stroke services. Any other adherence queries will be managed between the research team, site champion and principal investigator on a case-by-case basis.

### Sample Size

No formal sample size calculation was conducted. As the primary aim is to evaluate feasibility, we aimed to recruit up to 80 stroke survivors across sites (an average of 40 participants per site), allowing for 25% attrition. These numbers are in line with UK averages on sample sizes for feasibility studies.^30^ Assuming 50%–60% of stroke survivors will have a carer/family member who can take part we further aimed to recruit 45 carers (N=45 target), and 15 healthcare professionals. There are no planned interim analyses.

### Harm Monitoring

There are no outcome measures for harm monitoring, however we will monitor deaths, recurrent stroke, cognitive decline and worsening emotional symptoms of consenting patients who had a stroke using a customised survey following the end of participation of the stroke participant, completed by the staff at recruited sites.

### Participant Retention Plan

The research team will give up to three reminders to study participants to complete outcome assessments via email or telephone call within a month of their 6-month assessment window.

### Data Management

Data will be entered securely by recruited sites on Research Electronic Data Capture data management software. The research team will regularly download and anonymise data from recruited sites into a single cohesive dataset. Data audits on 10% of study data will be carried out to ensure data quality.

### Data Monitoring

An ENRICH advisory committee, comprising a PCPI representative and complex intervention and trial statistician expertise, will meet approximately triannually to oversee progress, advise on trial conduct and methodology.

### Quantitative Analysis

All quantitative data will be reported descriptively and will include all recruited participants. Rates of recruitment (numbers recruited per month, total numbers recruited, recruitment across participant categories), retention of sites and participants, number of ENRICH reviews delivered and number of patients receiving ENRICH reviews within the stroke service will be reported. Missing data will not be imputed.

Data on demographics (eg, age, sex, ethnicity, stroke severity) and study outcome measures at the post-ENRICH research visit will be summarised using descriptive statistics. Comparison of our recruited cohort to Sentinel Stroke National Audit Programme data, a UK quality improvement programme capturing national stroke outcomes, will be conducted to determine the generalisability of our cohort findings to the UK stroke population.

### Qualitative Analysis

Results from the post-study survey will be summarised to determine whether patients who had a stroke and carers found the enhanced reviews helpful, relevant and whether information needs regarding post-stroke psychological needs were sufficiently met. We will similarly describe average ratings of helpfulness of ENRICH reviews from recruited healthcare professionals. Any changes required prior to the progression of a definitive trial based on acceptability data will be reported.

Qualitative data will be analysed using a framework analysis based on the Theoretical Framework of Acceptability (TFA^31^). The aim of the qualitative analysis will be to understand why and in what specific circumstances the intervention did or did not work and to identify potential ways to improve the intervention as a means of informing intervention acceptability. The research team will meet regularly throughout the transcription process to revise the analytical framework. Once all transcripts are coded, data will be charted in a framework matrix with candidate codes and participant quotes labelled by identification number, mapped against TFA components of affective attitude, burden, perceived effectiveness, ethicality, intervention coherence, opportunity costs and self-efficacy. The research team will reflect on the matrix and adapt it to represent other units of analysis (eg, clinicians vs participants, participants with cognitive impairment vs participants without cognitive impairment) to develop an understanding of intergroup similarities and differences in intervention perceptions across TFA domains. A final theme structure will then be constructed from the matrices that describes key barriers and facilitators to intervention delivery and potential modifications, if any, to be made for a future trial. In combination with helpfulness and usefulness ratings from participants, we will define acceptability as average helpfulness and usefulness ratings of at least 3.5 out of 5 on our custom survey and a smaller number of barriers reported in our framework analysis compared with perceived intervention benefits from patients who had a stroke and carers.

### Fidelity Analysis

Healthcare professional-reported fidelity checklists will be summarised descriptively to determine average adherence to the intervention, specific intervention components delivered and reasons for non-adherence.

Approximately 20% of audio recorded ENRICH review sessions will be selected to rate for in-depth fidelity of ENRICH intervention components (table 2). The research team will evaluate fidelity at critical time windows (eg, when conducting early reviews following training; after having conducted several ENRICH reviews) and prespecify recorded sessions to be evaluated for fidelity based on purposive sampling on psychological profiling (ensuring a range of severity, and whether fidelity varies based on patient profile). Recordings will be listened to by the research team and scored for presence of key components described above as well as quality of delivery. The research team will additionally evaluate proportion of time spent on intervention factors (eg, the content of the ENRICH reviews) versus factors unrelated to ENRICH (eg, social conversation).

### Study Progression Criteria

A priori defined progression criteria (table 4) will be used to determine whether the intervention can proceed to testing effectiveness in a cluster randomised controlled trial. These criteria were selected based on feasibility objectives, clinical assumptions and pragmatic expectations. If we do not meet targets in green, indicating that modifications are needed prior to progressing to an effectiveness trial, we will identify potential modifications from interview data described above with stroke participants, carers and healthcare professionals involved in ENRICH and agree on suitable modifications with the ENRICH advisory and PCPI committees. Final modifications will be discussed with the ENRICH advisory committee.

**Table 4:** Progression criteria for ENRICH to a future effectiveness trial

Evaluation domain	Evaluation criterion	Green Proceed to trial	Amber Proceed to trial after modifications	Red Do not proceed to trial
Feasibility	Site recruitment	Two sites recruited within 6 months of REC approval.	Two sites recruited in more than 6 months since REC approval.	Unable to recruit sites.
	HCP recruitment	>50% of HCPs trained consented.	25%–50% of HCPs trained consented.	Less than 25% HCPs trained consented.
	Patients who had a stroke recruitment	Average >5 patients recruited per month.	Average 3–5 patients recruited per month.	<3 patients recruited per month by a site.
	Family member recruitment	>30% of recruited participants will have a family member who consent to provide outcome data.	10%–30% of recruited patients have a family member who consent to provide outcome data.	<10% of recruited patients have a family member who consent to provide outcome data.
Acceptability	ENRICH intervention delivery	≥50% of participants complete >1 ENRICH review.	30%–50% of participants complete >1 ENRICH review.	<30% of participants complete >1 ENRICH review.
	Outcome measure completion	≥75% completion of primary outcome measures (≥75%).	Moderate rates of completion of primary outcome measures (≥50%–75%).	Low rates of completion of primary outcome measures (<50%).
	Patients who had a stroke acceptability	Average rating of >3.5 out of 5 for helpfulness of ENRICH reviews.	Average rating of 3 out of 5 for helpfulness of ENRICH reviews.	Average rating less than 3 out of 5 for helpfulness of ENRICH reviews.
	Intervention delivery	Qualitative data supports feasibility with minor modifications.	Qualitative data supports feasibility but with major modifications made.	Qualitative data does not support feasibility, even with major modifications.
Fidelity	Treatment fidelity	≥70% adherence ratings across sites.	50%–70% treatment fidelity across sites.	Less than 50% treatment fidelity across sites.

ENRICH, ENhanced Reviews of PsychologIcal Changes; HCP, Health Care Professional; REC, Research Ethics Committee.

## Ethics and dissemination

This study was approved by the London Brighton and Sussex Research Ethics Committee (LO/24/0341). Protocol amendments will be communicated to relevant parties per local ethical standards. The study was prospectively registered on ISRCTN (ISRCTN16018388) where the protocol and analysis plan can be accessed. We will openly share anonymised data and statistical code from feasibility study results. All participants will provide written or verbal informed consent, or have positive consultee advice for research data collection, in accordance with the Declaration of Helsinki. The ENRICH study will be disseminated widely at stroke and healthcare conferences, through peer-reviewed academic publications and lay newsletters and summaries to participants and to the public. We will hold an end-of-study event to share study results with study participants.

### Implications and summary

Psychological changes following stroke are recommended to be evaluated and treated by stroke UK clinical guidelines. Despite this, there is variation across the UK in terms of psychological care received, with some parts of the UK not having any psychological care provision, resulting in unmet needs that last well into the long-term post-stroke.

The ENRICH feasibility study will provide important information on intervention delivery within the stroke care pathway under real-world conditions. Having broad inclusion criteria, having a waiver of consent for the ENRICH intervention and collecting site-level information about participation maximises information we can use to inform a decision to move to a definitive cluster trial or whether alternative trial methods are needed following identification of modifications (eg, individual randomisation). Importantly, this study will evaluate the acceptability and feasibility of this novel, co-designed intervention across a wide range of healthcare professionals, highlighting its potential to broaden access to psychological care more generally. Collecting information on training needs and barriers and facilitators to intervention delivery across such a diverse healthcare professional profile will be critical for translating ENRICH into a larger number of stroke services for an effectiveness trial to formally evaluate impact on post-stroke quality of life.
