# Supplementary material for: Feasibility and acceptability of ENhanced Reviews of PsychologIcal Changes (ENRICH) after stroke: protocol
Source: BMJ Open. 2026 May 24;16(5):e116566. doi: 10.1136/bmjopen-2026-116566 (PMC13202136; doi:10.1136/bmjopen-2026-116566)
Supplement: Supplementary file 1 [file bmjopen-2026-116566.reviewer_comments.pdf]

PEER REVIEW HISTORY

BMJ Open publishes all reviews undertaken for accepted manuscripts. Reviewers are asked to complete a checklist review form and are provided with free text boxes to elaborate on their assessment. These free text comments are reproduced below.

ARTICLE DETAILS

Title (Provisional)

Feasibility and Acceptability of ENhanced Reviews of PsychologIcal Changes (ENRICH) after stroke: PROTOCOL

Authors

Kusec, Andrea; Hobden, Georgina; Wright-Hughes, Alexandra; Demeyere, Nele

---

VERSION 1 - REVIEW

**Reviewer**	**1**
**Name**	**Hinwood, Madeleine**
**Affiliation**	**The University of Newcastle, School of Medicine and Public Health**
**Date**	**10-Mar-2026**
**COI**	**None**

This protocol describes the feasibility evaluation of ENRICH, a co-designed intervention to improve recognition and management of post-stroke psychological changes, including cognition, mood, and fatigue. The topic is clinically important and highly relevant to gaps in current stroke care. I have some minor comments only, which should be addressed for the protocol to be reproducible and easy for readers to interpret:

There is some confusion for readers in the current reporting of assessment schedules and time points. e.g. the abstract says stroke patients and carers will complete questionnaires at 6 and 9 months post-stroke. However, Table 3 presents Baseline (T1) and Post-ENRICH assessment (T2), and the quantitative analysis section later refers to post-ENRICH and at 3-month follow up. In addition, the text states that candidate outcomes were not collected at baseline, which appears inconsistent with Table 3. I think one clear and consistent schedule of enrolment, intervention delivery, and outcome assessment, used identically in the abstract, methods, tables, and analysis sections are required, with consistent terminology throughout for the timepoints used.

The study design is described in slightly different ways throughout, including a single arm multicentre feasibility study, a single-arm cluster design, a non-randomised study across two sites, and a study delivered at the site/cluster level. These descriptions are related, but the

protocol would benefit from one standard formulation early in the methods section, followed by a short explanation of what is clustered and what is not. As it reads, the design description feels more complex than necessary and risks confusing readers.

The ethical and methodological rationale for the waiver of consent is an important aspect of the proposed study that needs to be clarified. The paper needs to clearly distinguish between consent for receiving ENRICH as a service level intervention and consent for providing research data- at present, this delineation is unclear to the naive reader. Arguing that ENRICH is in line with minimum stroke standards is not strong enough. I think you need to include additional information around this including what elements are covered by the waiver, how patients lacking capacity are protected, what data are collected on patients who do not consent to having research data collected, and how it aligns with the service-level nature of the intervention.

Double check that feasibility and acceptability outcomes are all well defined (enough to enable replication)- e.g. what constitutes completion of an ENRICH review, what is sufficient to state intervention participation is complete, denomintaor for rates of review delivery, how constructs such as helpfulness and usefulness are to be measured.

VERSION 1 - AUTHOR RESPONSE

Reviewer 1

1) This protocol describes the feasibility evaluation of ENRICH, a co-designed intervention to improve recognition and management of post-stroke psychological changes, including cognition, mood, and fatigue. The topic is clinically important and highly relevant to gaps in current stroke care. I have some minor comments only, which should be addressed for the protocol to be reproducible and easy for readers to interpret.

Thank you for your positive evaluation of our work.

2) There is some confusion for readers in the current reporting of assessment schedules and time points. e.g. the abstract says stroke patients and carers will complete questionnaires at 6 and 9 months post-stroke. However, Table 3 presents Baseline (T1) and Post-ENRICH assessment (T2), and the quantitative analysis section later refers to post-ENRICH and at 3-month follow up. In addition, the text states that candidate outcomes were not collected at baseline, which appears inconsistent with Table 3. I think one clear and consistent schedule of enrolment, intervention delivery, and outcome assessment, used identically in the abstract, methods, tables, and analysis sections are required, with consistent terminology throughout for the timepoints used.

Thank you for your careful eye, we previously had a 9-month post-stroke follow-up assessment (i.e. a second research visit) but this was removed to prioritise the more in-depth information collected at the 6-month timepoint and outcome research visit. We have revised the text to make our terminology consistent in the abstract and throughout manuscript, some examples are below:

"Stroke patients and carers will complete demographics at baseline (T1) and questionnaires of quality of life, mood, and healthcare resource use at 6-months post-stroke (T2)" (page 2)

"Stroke participant demographic data will be collected prior to the first ENRICH review at baseline (T1)" (page 13)

"…study outcome measures at the post-ENRICH assessment session will be summarised using descriptive statistics" (page 21)

Table 3 additionally has checkmarks to indicate when each measure is being collected, with a checkmark for T1 (baseline) only for Demographic information.

3) The study design is described in slightly different ways throughout, including a single arm multicentre feasibility study, a single-arm cluster design, a non-randomised study across two sites, and a study delivered at the site/cluster level. These descriptions are related, but the protocol would benefit from one standard formulation early in the methods section, followed by a short explanation of what is clustered and what is not. As it reads, the design description feels more complex than necessary and risks confusing readers.

Thank you for this comment. We have revised the text and now use the terminology "single arm multicentre feasibility study" throughout the manuscript for consistency. We have additionally clarified in our methods section what is meant by "delivered at the site/cluster level":

"We considered a site to be at least 1 NHS stroke service comprising the full stroke care pathway from acute to 6-months post-stroke. Where this timeline two separate services, this was considered 1 site for the purposes of delivering the ENRICH intervention." (page 5)

4) The ethical and methodological rationale for the waiver of consent is an important aspect of the proposed study that needs to be clarified. The paper needs to clearly distinguish between consent for receiving ENRICH as a service level intervention and consent for providing research data- at present, this delineation is unclear to the naive reader.

Thank you. We have amended the Methods section in several places to clarify that the waiver of consent is for the intervention only, and that written informed consent/consultee advice procedures are in place for research data collection, with examples below:

"*We include a waiver of consent for the ENRICH intervention delivery only*." (page 6)

"*Taken together, we aimed to investigate acceptability and feasibility of the intervention delivery, as well as design feasibility of a future cluster design including waiver of consent for delivery of the ENRICH intervention*" (page 8)

"*The below outcome measures will be completed by stroke patients consenting to research data collection stroke patients only*" (page 15)

"*Stroke patient and carer participants consenting to research data collection will be invited to complete a custom post-study survey about their experience of ENRICH reviews at T2*" (page 19)

"*All participants will provide written or verbal informed consent, or have positive consultee advice for research data collection, in accordance with the Declaration of Helsinki*." (page 26)

5) Arguing that ENRICH is in line with minimum stroke standards is not strong enough. I think you need to include additional information around this including what elements are covered by the waiver, how patients lacking capacity are protected, what data are collected on patients who do not consent to having research data collected, and how it aligns with the service-level nature of the intervention.

We have revised the text to be clearer about the ethical aspects of the waiver of consent within the ENRICH research study. We have expanded our manuscript, as below:

"*The waiver of consent covers the delivery of the full ENRICH intervention delivered at 1,3 and 6 months post-stroke, including conducting the assessments, communicating screening results, and self-management of psychological changes (see ENRICH Intervention below for further specifics). A single-arm multicentre feasibility study design with a waiver of consent for intervention delivery was selected because 1) ENRICH was designed to be implemented for all stroke patients in a service as a standard of psychological care; 2) we aim to evaluate the ENRICH intervention as a service change for all, rather than an intervention for only a specific subset of stroke patients, and 3) there was deemed a potential for contamination within a service where ENRICH-trained health care professionals typically jointly discuss stroke patient care, thereby also limiting the potential for evaluation within an individually randomised controlled trial.*

The waiver of consent does not cover transfer of any research data about the ENRICH assessments or identifiable patient demographics to the research team outside of the patient's NHS service. Written informed consent or favourable consultee advice is required to be in place for this research data collection to occur. Only fully anonymised site-level information will be collected about total number of patients in the recruited site who had received an ENRICH review during the study timeframe. Patients lacking capacity will be protected through having consultee advice procedures in place, conducting with a family member who knows the patient well, to advise on what they believe the patient would have wanted with regards to research data collection." (pages 6-7)

6) Double check that feasibility and acceptability outcomes are all well-defined (enough to enable replication)- e.g. what constitutes completion of an ENRICH review, what is sufficient to state intervention participation is complete, denominator for rates of review delivery, how constructs such as helpfulness and usefulness are to be measured.

We have now defined our quantitative feasibility and acceptability outcomes more clearly and explicitly in our Methods section (pages 17 – 24) – some examples are copied below:

"*With regards to study design, quantitative data on number of sites meeting initial site eligibility and numbers of expressions of interest will be collected. Per site, participant eligibility rates (defined as percentage of patients per site meeting study eligibility criteria), recruitment rates (total number of patients recruited per month across both informed and consultee advised consent), intervention completion (total number of ENRICH reviews received) and outcome completion rates for stroke patients, carers (T2 assessment completion rates), and health care professionals (interview completion) will be collected.*"*(*page 17)

"Our quantitative aims with regards to the delivery of the intervention are to investigate the numbers of trained clinicians delivering ENRICH reviews, proportions of recruited patients receiving ENRICH reviews, and ratings of perceived helpfulness and usefulness (assessed using a custom Likert scale from 1 – 5) of ENRICH from health care professionals, stroke patients, and carers." (page 18)

"We aim to assess completion rates of candidate outcome measures (i.e., total proportion of participants completing each outcome measure) at T2 to inform acceptability." (page 18)

"*In combination with helpfulness and usefulness ratings from participants, we will define acceptability as average helpfulness and usefulness ratings of at least 3.5 out of 5 on our custom survey and a smaller number of barriers reported in our qualitative framework analysis compared to perceived intervention benefits from stroke patients and carers*." (page 24).

VERSION 2 - REVIEW

**Reviewer**	**1**
**Name**	**Hinwood, Madeleine**
**Affiliation**	**The University of Newcastle, School of Medicine and Public Health**
**Date**	**24-Apr-2026**
**COI**	

All prior comments have been addressed satisfactorily. The protocol is clear, internally consistent, and sufficiently detailed to support reproducibility.
